# A Systematic Review on the Diagnosis of Pediatric Bacterial Pneumonia: When Gold Is Bronze

**DOI:** 10.1371/journal.pone.0011989

**Published:** 2010-08-06

**Authors:** Tim Lynch, Liza Bialy, James D. Kellner, Martin H. Osmond, Terry P. Klassen, Tamara Durec, Robin Leicht, David W. Johnson

**Affiliations:** 1 Department of Pediatrics, Children's Hospital, University of Western Ontario, London, Ontario, Canada; 2 Department of Pediatrics, University of Alberta, Edmonton, Alberta, Canada; 3 Department of Pediatrics and Physiology and Pharmacology, University of Calgary and Alberta Children's Hospital, Calgary, Alberta, Canada; 4 Clinical Research Unit, Children's Hospital of Eastern Ontario Research Institute, Ottawa, Ontario, Canada; Universidad Nacional Mayor de San Marcos, Peru

## Abstract

**Background:**

In developing countries, pneumonia is one of the leading causes of death in children under five years of age and hence timely and accurate diagnosis is critical. In North America, pneumonia is also a common source of childhood morbidity and occasionally mortality. Clinicians traditionally have used the chest radiograph as the gold standard in the diagnosis of pneumonia, but they are becoming increasingly aware that it is not ideal. Numerous studies have shown that chest radiography findings lack precision in defining the etiology of childhood pneumonia. There is no single test that reliably distinguishes bacterial from non-bacterial causes. These factors have resulted in clinicians historically using a combination of physical signs and chest radiographs as a ‘gold standard’, though this combination of tests has been shown to be imperfect for diagnosis and assigning treatment. The objectives of this systematic review are to: 1) identify and categorize studies that have used single or multiple tests as a gold standard for assessing accuracy of other tests, and 2) given the ‘gold standard’ used, determine the accuracy of these other tests for diagnosing childhood bacterial pneumonia.

**Methods and Findings:**

Search strategies were developed using a combination of subject headings and keywords adapted for 18 electronic bibliographic databases from inception to May 2008. Published studies were included if they: 1) included children one month to 18 years of age, 2) provided sufficient data regarding diagnostic accuracy to construct a 2×2 table, and 3) assessed the accuracy of one or more index tests as compared with other test(s) used as a ‘gold standard’. The literature search revealed 5,989 references of which 256 were screened for inclusion, resulting in 25 studies that satisfied all inclusion criteria. The studies examined a range of bacterium types and assessed the accuracy of several combinations of diagnostic tests. Eleven different gold standards were studied in the 25 included studies. Criterion validity was calculated for fourteen different index tests using eleven different gold standards. The most common gold standard utilized was blood culture tests used in six studies. Fourteen different tests were measured as index tests. PCT was the most common measured in five studies each with a different gold standard.

**Conclusions:**

We have found that studies assessing the diagnostic accuracy of clinical, radiological, and laboratory tests for bacterial childhood pneumonia have used a heterogeneous group of gold standards, and found, at least in part because of this, that index tests have widely different accuracies. These findings highlight the need for identifying a widely accepted gold standard for diagnosis of bacterial pneumonia in children.

## Introduction

In developing countries, pneumonia is one of the leading causes of death in children under five years of age and hence timely and accurate diagnosis is critical [1]. In North America, pneumonia is also a common source of childhood morbidity and occasionally mortality [2]. A study from Israel has also shown that there can be significant economic burdens to children and families dealing with community acquired pneumonia, as well as significant reduction in their quality of life [3].

Viruses, atypical, and typical bacteria cause the vast majority of childhood pneumonia [2]–[4] The distribution of pathogens varies with age and clinical setting. Atypical bacterial microorganisms, such as Mycoplasma and Chlamydia usually occur in children between the ages of five and 15 years [5]–[7], while the incidence of viral infections typically decreases with age [5]. In hospitalized children, the most frequently diagnosed bacteria are the typical pathogens, such as Streptococcus pneumoniae [5]. It can be difficult to identify whether the cause of pneumonia in a given patient is bacterial or nonbacterial [8], [9]. Classic signs unique to bacterial or nonbacterial pneumonia can be helpful in coming to a diagnosis [9]. However, these signs and symptoms are often subjective, and are ultimately imprecise at determining whether antibiotics are truly warranted [4].

A clinically acceptable gold standard for the diagnosis of bacterial pneumonia has not yet been developed [2], [5], [8]. Often the most readily available means of diagnosing pneumonia are through observations of physical signs and radiological evidence. Diagnostic guidelines have been developed by the World Health Organization for pneumonia and these are generally used in developing countries or in the absence of quick access to laboratory tests [10]. Other diagnostic tests have been used with variable rates of accuracy, such as chest radiographs, laboratory tests (white blood cell count [WBC]) with differential, C-reactive protein (CRP), erythrocyte sedimentation rate (ESR) [8], [9], blood cultures and serology [8], and lung puncture [8], [9]. The ideal surrogate marker for bacterial pneumonia should be accurate, minimally invasive, and readily available. To date, there is no such gold standard that a physician can rely on to confidently diagnose and subsequently treat bacterial pneumonia [2].

Clinicians traditionally have used the chest radiograph as the gold standard in the diagnosis of pneumonia, but they are becoming increasingly aware that it is not ideal. Numerous studies have shown that chest radiography findings lack accuracy in defining the etiology of childhood pneumonia [2], [11], [12]. There is no single test that reliably distinguishes bacterial from non-bacterial causes [4]. These factors have resulted in clinicians historically using a combination of physical signs and chest radiographs as a ‘gold standard’, though this combination of tests has been shown to be imperfect for diagnosis and assigning treatment [5], [13].

The objectives of this systematic review are to: 1) identify and categorize studies that have used single or multiple tests as a gold standard for assessing accuracy of other tests, and 2) given the ‘gold standard’ used, determine the accuracy of these other tests for diagnosing childhood bacterial pneumonia.

## Methods

This review has been carried out using methods defined for rigorous systematic reviews [14], [15]. The aim was to use these guidelines and other methodological criteria [16]–[18] to produce a systematic review that is comprehensive and summarizes the data collected (see PRISMA Checklist S1).

### Ethics Statement

Data for this study was acquired through previously published work, no patient or hospital data was accessed. Therefore, written consent and institutional ethical review was not required for this research.

### Search Strategy and Selection Criteria

Search strategies were developed using a combination of subject headings and keywords, including: “pneumonia”, “bacteria”, “community acquired pneumonia”, “lower respiratory tract infection”, “pneumococcal”, “diagnosis”, “accuracy”, “sensitivity”, “reliability”, “specificity”, “false/true positive/negative”, “predictive value”, “observer variation”, “likelihood functions/ratios”, “ROC curve”, “receiver operating characteristic”, “child”, “adolescent”, “infant”, “minors”, “pediatrics”, “nurseries”, “youth”, “nursery”, “nurseries”, “toddler”, “clinical trials”, “cohort studies”, “case-control studies”, “comparative”, “evaluation studies”, “prospective”, “retrospective”, and “follow up”.

These keywords were adapted for each of the 18 electronic bibliographic databases from inception to May 2008 (see Table 1 for full listing). Extended systematic search methods (e.g., hand searches of non-indexed journals, reference list tracking, and contact with experts) were also used (See Table 2 for full listing). No language or date restrictions were applied to the search strategy.

**Table 1:** Databases and Trials Registers Included in Search.

Source/Database Name	Source/Year
MEDLINE®	(Ovid; 1950–April 2008)
Ovid Medline® In-Process & Other Non-Indexed Citations	(up to April 2008)
Cochrane Central Register of Controlled Trials	(Ovid; 1^st^ Quarter, 2008)
Cochrane Database of Systematic Reviews	(Ovid; 1^st^ Quarter, 2008)
Database of Abstracts of Reviews of Effects (DARE)	(Ovid; 1^st^ Quarter 2008)
EMBASE®	(1988–April 2008)
CINAHL	(EBSCOhost; 1937–April 2008)
HealthSTAR	(Ovid; 1966–April 2008)
Global Health	(Ovid; 1987–April 2008)
Pascal	(Ovid; 1987–April 2008)
BIOSIS Previews®	(via Web of Science® ; 1969–April 2008)
Science Citation Index Expanded™ and Social Science Citation Index®	(via Web of Science®; 1900–April 2008)
PubMed	(1966 to October 2006)
Current Controlled Trials, ClinicalTrials.gov, Clinical Trials in Cardiology	http://www.controlled-trials.com/, http://clinicaltrials.gov/, www2.umdnj.edu/~shindler/trials/trials_a.html
National Research Register	www.update-software.com/National/
Computer Retrieval of Information on Scientific Projects (CRISP)	http://crisp.cit.nih.gov
Australian Clinical Trials Registry	www.actr.org.au
MEDION	www.mediondatabase.nl
NLM (National Library of Medicine) Gateway, BioMed Central, and OCLC PapersFirst were searched for identification of meeting abstracts	

**Table 2:** Grey Literature Databases/Websites Searched.

Website	URL
Agency for Healthcare Research and Quality (AHRQ)	http://ahcpr.gov
American Academy of Allergy Asthma & Immunology	http://www.aaaai.org/members/annual_meeting
American Academy of Pediatrics	http://www.aap.org/
American College of Chest Physicians	http://www.chestnet.org/
American Thoracic Society	http://www.thoracic.org/sections/meetings-and-courses/index.html
Annual Meeting of the European Society for Paediatric Infectious Diseases (ESPID)	http://www.espid.net/
Annual Meeting of the Infectious Diseases Society of America (IDSA) 2001–2005	http://www.idsociety.org/
Asian Pacific Society of Respirology, 11th APSR Congress	http://www.apsresp.org/
Australian Clinical Trials Registry	http://www.actr.org.au/
Basal Institute of Clinical Epidemiology	http://www.bice.ch/engl/research.htm
Basel Institute for Clinical Epidemiology	http://www.bice.ch/engl/home.htm
Bayes Library of diagnostic Studies and Reviews	http://www.ispm.ch
British Thoracic Society	http://www.brit-thoracic.org.uk/
Conference on Global Lung Health	http://www.worldlunghealth.org/
Database of Promoting Health Effectiveness Reviews (DoPHER)	http://eppi.ioe.ac.uk.cms
Database of Systematic Reviews in Clinical Chemistry and Laboratory Medicine	http://www.ckcjl-mb.nl/ifcc/
European Congress of Clinical Microbiology and Infectious Diseases 2001–2005	http://www.akm.cc/eccmid2001-2005/
European Respiratory Society	http://dev.ersnet.org/
European Society of Clinical Microbiology and Infectious Diseases (ESCMID)	http://www.escmid.org/
GreyLit Report	http://nyam.org/library/grey.shtml
Guidelines International Network	http://www.g-i-n.net
Health Evidence	http://hebs.cf.ac.uk
Health Technology Assessment Database	http://agatha.york.ac.uk/htahp.htm
HTAi vortal Health Technology Assessment International	http://www.htai.org/vortal/
Infectious Diseases Society of America	http://www.idsociety.org/
International Pediatric Association	http://www.ipa-world.org/meetings/meetings.htm
International Society for Infectious Diseases	http://www.isid.org/
Interscience Conference Antimicrobial Agents and Chemotherapy	http://www.icaac.org
LWWOnline: The Pediatric Infectious Disease Journal	http://pidj.com
MEDION	http://www.mediondatabase.nl/
National Institute of Allergy and Infectious Diseases (NIAID)	http://www3.niaid.nih.gov
New York Academy of Medicine Library, Grey Literature Collection	http://www.nyam.org/library/grey.shtml
Oxford Childhood Infection Study	http://www.dphpc.ox.ac.uk/oxcis/
Pediatric Academic Societies Archive 2000–2005	http://www.abstracts2view.com/pasall
Pediatric Critical Care Medicine	http://www.pccmjournal.com
The Society for Clinical Trials	http://www.sctweb.org/
US Department of Health and Human Services, Centers for Disease Control and Prevention	http://www.cdc.gov/ncidod/
World Health Organization (WHO)	http://search.who.int
World Congress on Pediatric Critical Care	http://www.wfpiccs.org/

Inclusion criteria were assessed independently by at least two reviewers (LB and RL). The primary reason for exclusion of articles was documented. Scientific-based publications were included if they: (1) involved children between the ages of 1 month and 18 years of age, (2) provided diagnostic accuracy data to construct a 2×2 table, and (3) compared a gold standard and index test that were both used to make a diagnosis of bacterial pneumonia taken to include both typical and atypical pneumonia. Gold standard and index test categories included radiographic, hematologic, immunologic, microbiologic, virologic, and clinical variables (signs and symptoms). Due to the lack of a defined gold standard that can reliably differentiate bacterial from non-bacterial pneumonia, all combinations of tests assessing the diagnostic accuracy of bacterial pneumonia were included. To assess study quality the Quality Assessment of Studies of Diagnostic Accuracy included in Systematic Reviews (QUADAS) was applied by two independent reviewers (LB and RL) [19], [20].

### Data Extraction and Analysis

Data was extracted by one reviewer and checked for accuracy and completeness by a second reviewer. Any disagreements were resolved through discussion with the clinical leaders.

Data analysis was based on a published methodological review [21]. The primary outcome is accuracy of the screening test (i.e., sensitivity, specificity, positive and/or negative predictive values with corresponding 95% confidence intervals using standard formulas) [22]. For each individual study, we reconstructed a standard 2×2 table and if multiple studies had used the same index test and gold standard weighted averages of the sensitivities, specificities or predictive values were computed.

## Results

**Figure 1:**
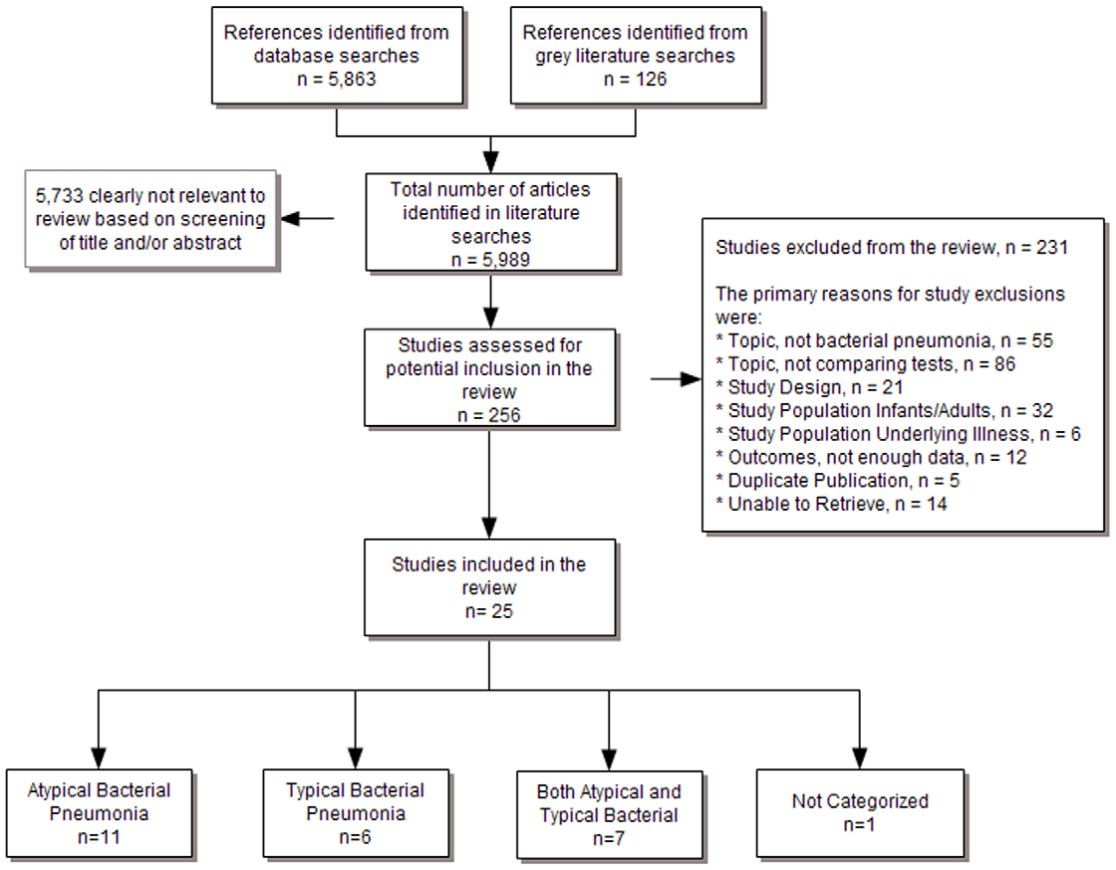
PRISMA flowchart of study selection, retrieval, and inclusion.

The literature search revealed 5,989 references of which 256 were screened for inclusion. As shown in Figure 1 this resulted in 25 studies that satisfied all inclusion criteria.

### Study Characteristics

The studies examined a range of bacterium types and assessed the accuracy of several combinations of diagnostic tests. Detailed characteristics of each study appear in Tables S1 and S2. These studies were published between 1986 and 2007 from 12 different countries. The majority of included studies originated from higher income countries (Australia, Italy, Spain, France, United States, Switzerland, Japan, and Finland), as defined by The World Bank, with 7 studies from the middle to low income category (China/Taiwan, Argentina, Brazil, and Bangladesh) [23]. All subjects were children between the ages of one month and 17 years, with a mean age of 6.56 years (based on 14 studies reporting a mean or median age). Gender was evenly distributed as specified in 12 of 25 studies (52.3% male). The majority of the studies collected patient data prospectively (21/25) from a single site (24/25). Eleven studies examined atypical species of bacterial pneumonia, six looked at typical bacteria, and seven combined both atypical and typical varieties. One study defined what they studied only as ‘bacterial pneumonia’.

To be included in our review studies needed to clearly describe both the index and gold standards used. A specific gold standard was not defined *a priori*, therefore all combinations of index tests and gold standards were included, provided the studies met all inclusion criteria. We broadly categorized the types of diagnostic tests (both gold and index) as radiographic, hematologic, immunologic, microbiologic, virologic, or clinical variables (signs/symptoms) for ease of comparison. From the 25 included articles, we ended up with 23 distinct combinations of these categories. As a result of the wide range of testing modalities it was not possible to combine studies or compute weighted accuracy data (see Table S3 for individual study tests and data). Therefore we conducted a qualitative review of this literature and non-numerically summarized the major findings. Results for each of the studies can be found in Table S3. All 25 articles were assessed using the QUADAS tool and the scores ranged from 8 to 14, with an average score of 10.44 (see Table S4 for quality assessment of individual studies).

### Categorization of Gold Standards

Eleven different gold standards were studied in the 25 included studies. The most common gold standard utilized was blood culture tests used in six studies [24]–[29]. These studies measured the criterion validity of nine different index tests, including the measurement of signs/symptoms, hematologic, chest radiograph, nested Polymerase Chain Reaction (PCR), procalcitonin (PCT), CRP, latex agglutination, immunochromatographic membrane assay, and lung aspirate. Sensitivities ranged from 10% for the lung aspirate as an index test to 100% with urine latex agglutination for Hib as an index test. Specificities ranged from 63.2% for the chest radiograph as an index test to100% with nested PCR as an index test.

Five studies [25], [30]–[33] used a chest radiograph either alone or with other variables as the gold standard, measuring the validity of seven index tests. These index tests included: PCT with three cutoff points, WBC count, CRP, serology by complement fixation in 2 studies, latex particle agglutination, and nested PCR. With the chest radiograph as the gold standard, sensitivities ranged from 14.3% (radiograph exhibited air trapping) to 77.8% (PCT>0.5 ng/ml) and specificities ranged from 34.8% (PCT>0.5 ng/ml) to 100% (nested PCR).

The one study [28] that used pleural fluid cultures as the gold standard revealed that the immunochromatographic membrane assay for urinary pneumococcal antigen detection had a sensitivity of 90.9% and a specificity of 68.8%. Other gold standards utilized in the studies included: hematologic [34], microbiologic [35]–[39], hematologic/immunologic [40], serology [41], [42], immunologic [43]–[46], and clinical signs and symptoms [47].

### Categorization of Index Tests

Fourteen different tests were measured as index tests. PCT was the most common measured in five studies [26], [30], [36], [42], [46], each with a different gold standard. There were as many as four separate cutoff points set for the PCT levels utilized. The sensitivity of PCT ranged from 40% when 0.5 ng/dl was set as the cutoff point and chest radiography was used as a gold standard to 95.4% in two studies with a cutoff above 0.5 ng/dl when blood cultures were used as the gold standard. For all studies its sensitivity decreased and its specificity increased as the cutoff points were raised.

An additional four studies used clinical variables, PCR, and CRP as their index tests. PCR was used as the index test [25], [34], [44], [45] with six different gold standards. Sensitivities ranged from 36.4% with complement fixation (Mycoplasma pneumonia) to 95.7% with Mycoplasma serology. Specificities ranged from 79.7% with mycoplasma serology to 100% with positive blood cultures or clinical and radiological evidence of pneumonia.

Clinical variables [24], [39]–[41] and CRP [36], [39], [46], [48] each demonstrated broad ranges in sensitivities and specificities for the array of clinical variables and the different cutoff points for CRP measured. The chest radiograph's accuracy was measured as an index test in four [24], [35], [40], [47] of the studies. Its sensitivity peaked at 75% with a range of 0–75% depending on the radiological definition assigned while its specificity ranged from 50 to 100%. Four studies [24], [39], [42], [48] utilized the total WBC count as an index test with three ranges being utilized. Sensitivities ranged from 20% to 65.1% and specificities ranged from 53.1% to79.3% when the total WBC count was above 15 000 (_X_10^6^/l).

Other index tests utilized in the studies included: interleukin-6 at 3 different levels [36], [42], [46], immunologic [28], [36]–[38], microbiologic [27], [31]–[33], [38], virologic [32], [43], hematologic [39], and lung aspirate [29].

## Discussion

Diagnostic testing provides physicians with information about the likelihood of certain diseases. Ideally these diagnostic tests have been validated against an agreed-upon gold/reference standard. The objective of the Standards for Reporting of Diagnostic Accuracy (STARD) initiative [49] is to improve the accuracy and completeness of reporting of studies of diagnostic accuracy. They defined the gold/reference standard to be “the best available method for establishing the presence or absence of the condition of interest.” They further add that “the reference standard can be a single method, or a combination of methods, to establish the presence of the target condition. It can include laboratory tests, imaging tests, and pathology, but also dedicated clinical follow-up of subjects.” This systematic review has demonstrated that diagnostic tests used for pediatric pneumonia have not been truly validated and there is little agreement as to what tests should be used as a gold standard. It is, therefore, difficult to recommend any of the reference standards used in the reviewed studies as “the best available method” given these limitations.

This review underscores the fundamental problem with diagnosing pneumonia in children when there is no proven and accurate gold standard. Since the standards used to define pneumonia are variable and inconsistent it is difficult to know whether the criterion validity of these diagnostic tests is accurate or not. A problem of ‘circularity’ exists for which there is no easy solution.

An additional problem is that the included studies did not all focus on the same type of bacterial disease. Eleven studies dealt with atypical pneumonia, six with typical pneumonia, and seven studied both typical and atypical pneumonia. And even within those studies which focused on the same type of bacterial etiology (e.g. pneumococcal pneumonia), each study defined the disease differently. For example, a patient with a positive blood culture for pneumococcus is likely clinically different from a patient with a negative blood culture.

One challenging aspect is that most of the studies were performed in high income countries with only seven studies performed in low income countries. This is contrast to the disease burden, where most of the mortality from pneumonia happens in low income countries. Future research should try to redress this imbalance.

### Categorization of Gold Standards

Of the eleven different gold standards utilized, the blood culture and the chest radiograph were the most common tests. Chest radiography was utilized in five studies as the gold standard while in three other studies it was measured as an index test. When it was employed as an index test its sensitivity was generally low while its specificity was generally high. This sub-par performance as an index test illustrates that the use of chest radiography as a gold standard is potentially flawed. The use of ten other gold standards for twenty of the studies highlights that there is much disagreement amongst researchers worldwide whether the chest radiograph should be utilized as a gold standard or an index test. In most academic emergency departments, a chest radiograph is considered the standard of care and is readily obtained for pediatric patients with the clinical suspicion of bacterial pneumonia. Clinically similar patients with potential ambulatory pneumonia presenting to a clinic or private office are less likely to undergo chest radiography.

From a clinical perspective, the blood culture is somewhat invasive and the results are generally not available for several hours. Further only a relatively small percentage of patients with bacterial pneumonia yield a positive blood culture (which results in low sensitivity), and now with the widespread use of conjugate pneumoococcal vaccine, the yield of blood cultures would be even less.

### Criterion Validity of Index Tests

Fourteen different index tests' criterion validity was measured. The heterogeneity of the different studies was further illustrated when the results of the different index tests were compared. Though the overall criterion validity of PCR was reasonably consistent, most other index tests (e.g. clinical variables, total WBC count, interleukin-6 and CRP) had highly variable accuracy.

As an example, in one study of PCT used as an index test, Don [30] concluded, in contrast to other studies, that serum PCT could not reliably distinguish bacterial from non-bacterial pneumonia. However, Don et al. [30] utilized as gold standard a chest radiograph that was inconclusive in 34% of their patients. This example illustrates that, given the diversity of the diagnostic methods used, current evidence is potentially inaccurate and highly misleading.

There is a critical need for experts in childhood pneumonia to develop an accepted gold standard. While it would be optimal for such a test to be cheap and readily available to practicing clinicians, the development of a more complex gold standard for use in research studies would be a major advance. As suggested in the STARD initiative,[49] one approach for developing a more complex standard is to use a combination of methods including imaging tests, laboratory tests available both immediately and long after the fact, and clinical features obtained not only at presentation but on dedicated follow-up subjects. The problem then becomes how each of these individual items should be weighted relative to the others. Given the highly variable results we found for most reference tests, a fixed algorithmic approach to combining methods is not possible. Alternatively an expert panel could use standard consensus methods to weigh the results of chest x-ray, standard and specialized laboratory tests, bacterial and viral diagnostic tests and clinical course of patients to classify patients as bacterial or non-bacterial.[50]–[51] The development of such a gold standard would greatly enhance and aid the evaluation of diagnostic tests for their accuracy in the future.

Although we conducted a comprehensive electronic and hand search of the literature, as well as verification of all extracted data this review is not without limitations. The main limitation of this review is the inability to include Latin American databases such as the Latin American and Caribbean Health Sciences Literature (LILACS) and Scientific Electronic Library Online (SciELO) as part of the electronic search strategy. At the onset of this review we were unable to identify a clinical expert fluent in Spanish to participate in the identification of search terms and in the screening, inclusion/exclusion, and extraction phases of the systematic review. African and Asian databases were also not included for similar reasons. We acknowledge this as a limiting factor of this review but with the breadth of other databases searched we do not believe this has altered the results.

In conclusion, we have found that studies assessing the diagnostic accuracy of clinical, radiological, and laboratory tests for bacterial childhood pneumonia have used a heterogeneous group of gold standards, and found, at least in part because of this, that index tests have widely different accuracies. These findings highlight the need for identifying a widely accepted gold standard for diagnosis of bacterial pneumonia in children.

## Supporting Information

Table S1 Study Characteristics.
(0.07 MB PDF)

Table S2 Participant Characteristics.
(0.06 MB PDF)

Table S3 Criterion Validity for the Diagnosis of Bacterial Pneumonia.
(0.06 MB PDF)

Table S4 Quality Assessment of Studies of Diagnostic Accuracy included in Systematic Reviews (QUADAS).
(0.01 MB PDF)

PRISMA Checklist S1 PRISMA Checklist of items to include when reporting a systematic review or meta-analysis (diagnostic review consisting of cohort studies).
(0.06 MB DOC)

## References

[1] Shann F, Steinhoff MC (1999). Vaccines for children in rich and poor countries.. Lancet.

[2] McIntosh K (2002). Community-acquired pneumonia in children.. N Engl J Med.

[3] Shoham Y, Dagan R, Givon-Lavi N, Liss Z, Shagan T (2005). Community-acquired pneumonia in children: quantifying the burden on patients and their families including decrease in quality of life.. Pediatrics.

[4] Michelow IC, Olsen K, Lozano J, Rollins NK, Duffy LB (2004). Epidemiology and clinical characteristics of community-acquired pneumonia in hospitalized children.. Pediatrics.

[5] McCracken GH (2000). Diagnosis and management of pneumonia in children.. Pediatr Infect Dis J.

[6] Ferwerda A, Moll HA, de Groot R (2001). Respiratory tract infections by Mycoplasma pneumoniae in children: a review of diagnostic and therapeutic measures.. Eur J Pediatr.

[7] Lichenstein R, Suggs AH, Campbell J (2003). Pediatric pneumonia.. Emerg Med Clin North Am.

[8] Coote N, McKenzie S (2000). Diagnosis and investigation of bacterial pneumonias.. Paediatr Respir Rev.

[9] Churgay CA (1996). The diagnosis and management of bacterial pneumonias in infants and children.. Prim Care.

[10] Benguigui Y, Antunano FJL, Schmunis G, Yunes J (1999). Respiratory infections in children. Pan American Health Organization..

[11] Lynch T, Gouin S, Larson C, Patenaude Y (2004). Does the lateral chest radiograph help pediatric emergency physicians diagnose pneumonia? A randomized clinical trial.. Acad Emerg Med.

[12] Wilkins TR, Wilkins RL (2005). Clinical and radiographic evidence of pneumonia.. Radiol Technol.

[13] Sinaniotis CA (1999). Community-acquired pneumonia: diagnosis and treatment.. Pediatr Pulmonol.

[14] Egger M, Davey Smith G, Altman DG (2001). Systematic reviews in health care: meta-analysis in context.

[15] Alderson P, Green S, Higgens JPT (2008). Cochrane Reviewers' Handbook4.2.2.. http://www.cochrane.org.

[16] Deville WL, Buntinx F, Bouter LM, Montori VM, de Vet HC (2002). Conducting systematic reviews of diagnostic studies: didactic guidelines.. BMC Med Res Methodol.

[17] Khan KS (2005). Systematic reviews of diagnostic tests: a guide to methods and application.. Best Pract Res Clin Obstet Gynaecol.

[18] Irwig L, Macaskill P, Glasziou P, Fahey M (1995). Meta-analytic methods for diagnostic test accuracy.. J Clin Epidemiol.

[19] Whiting P, Rutjes AW, Reitsma JB, Bossuyt PM, Kleijnen N (2003). The development of QUADAS: a tool for the quality assessment of studies of diagnostic accuracy included in systematic reviews.. BMC Med Res Methodol.

[20] Whiting PF, Weswood ME, Rutjes AW, Reitsma JB, Bossuyt (2006). Evaluation of QUADAS, a tool for the quality assessment of diagnostic accuracy studies.. BMC Med Res Methodol.

[21] Deeks JJ (2001). Systematic reviews in health care: Systematic reviews of evaluations of diagnostic and screening tests.. BMJ.

[22] Rosner B (1995). Fundamentals of biostatistics.

[23] The WorldBank. Data and Statistics: Country Classification (2009). The World Bank Group, editor.. http://web.worldbank.org/WBSITE/EXTERNAL/DATASTATISTICS/0,,contentMDK:20420458~menuPK:64133156~pagePK:64133150~piPK:64133175~theSitePK:239419,00.htm.

[24] Bettenay FA, de Campo JF, McCrossin DB (1988). Differentiating bacterial from viral pneumonias in children.. Pediatr Radiol.

[25] Mayoral C, Norona M, Baroni MR, Giani R, Zalazar F (2005). Evaluation of a nested-PCR assay for Streptococcus pneumoniae detection in pediatric patients with community-acquired pneumonia.. Rev Argent Microbiol.

[26] Prat C, Dominguez J, Rodrigo C, Gimenez M, Azuara M (2003). Procalcitonin, C-reactive protein and leukocyte count in children with lower respiratory tract infection.. Pediatr Infect Dis J.

[27] Saha SK, Baqui AH, El Areefin S, Qazi S, Billal DS (2006). Detection of antigenuria for diagnosis of invasive Haemophilus influenzae type b disease.. Ann Trop Paediatr.

[28] Tzeng DH, Lee YL, Lin YH, Tsai CA, Shi ZY (2006). Diagnostic value of the Binax NOW assay for identifying a pneumococcal etiology in patients with respiratory tract infection.. J Microbiol Immunol Infect.

[29] Vuori-Holopainen E, Salo E, Saxen H, Hedman K, Hyypia T (2000). Etiological diagnosis of childhood pneumonia by use of transthoracic needle aspiration and modern microbiological methods.[see comment].. Clin Infect Dis.

[30] Don M, Valent F, Korppi M, Falleti E, De Candia A (2007). Efficacy of serum procalcitonin in evaluating severity of community-acquired pneumonia in childhood.. Scand J Infect Dis.

[31] Jimenez AJ, Esteban RE, Girones IG, Sanchez PG, Orozco AL (1997). Usefulness of etiologic studies in pediatric inpatients with pneumonia.. An Esp Pediatr.

[32] Esteban RE, Jimenez AM, Orozco AL, Girones IG, Sanchez PG (1996). Etiology of acute respiratory infections in 87 hospitalized children.. Rev Clin Esp.

[33] Nunes AA, Camargos PA, Costa PR, Campos MT (2004). Antigen detection for the diagnosis of pneumonia.. Pediatr Pulmonol.

[34] Blackmore TK, Reznikov M, Gordon DL (1995). Clinical utility of the polymerase chain reaction to diagnose Mycoplasma pneumoniae infection.. Pathology (Phila).

[35] Castriota-Scanderbeg A, Popolizio T, Sacco M, Coppi M, Scarale MG (1995). Diagnosis of mycoplasma pneumonia in children: which is the role of thoracic radiography?. Radiol Med.

[36] Gendrel D, Moulin F, Lorrot M, Marc E, Gueren S (2002). Procalcitonin and markers of infection in community-acquired pneumonia of children.. Medecine et Maladies Infectieuses Editions Scientifiques et Medicales Elsevier SAS, Paris, France.

[37] Nagayama Y, Sakurai N, Yamamoto K, Honda A, Makuta M (1988). Isolation of Mycoplasma pneumoniae from children with lower-respiratory-tract infections.. J Infect Dis.

[38] Requejo HI (2007). Community-acquired pneumonia in the childhood: analysis of the diagnostic methods.. Braz J Infect Dis.

[39] Tsai M, Huang Y, Chen C, Lin P, Chang LY (2005). Chlamydial pneumonia in children requiring hospitalization: effect of mixed infection on clinical outcome.. Journal of Microbiology, Immunology and Infection Scientific Communications International Limited, Hong Kong, China.

[40] Esposito S, Bosis S, Cavagna R, Faelli N, Begliatti E (2002). Characteristics of Streptococcus pneumoniae and atypical bacterial infections in children 2–5 years of age with community-acquired pneumonia.. Clin Infect Dis.

[41] Gambert C, Werner E, de KM, Monginet F, Bruel H (1993). Mycoplasma pneumoniae pneumopathies in children: clinical, biological and radiological study.. Pediatrie.

[42] Moulin F, Raymond J, Lorrot M, Marc E, Coste J (2001). Procalcitonin in children admitted to hospital with community acquired pneumonia.. Arch Dis Child.

[43] Hardy RD, Michelow IC, Rios AM, Olsen K, Lozano J (2003). Comparison of nasopharyngeal and oropharyngeal PCR for the diagnosis of mycoplasma pneumonia in children.. 43rd ICAAC Abstracts (Abstract D-1859).

[44] Liu FC, Chen PY, Huang F, Tsai CR, Lee CY (2007). Rapid diagnosis of Mycoplasma pneumoniae infection in children by polymerase chain reaction.. J Microbiol Immunol Infect.

[45] Nadal D, Bossart W, Zucol F, Berger C, Lips R (1999). Mycoplasma pneumoniae pneumonia in children and its etiologic diagnosis.. Abstr Intersci Conf Antimicrob Agents Chemother Intersci Conf Antimicrob Agents Chemother.

[46] Toikka P, Irjala K, Juven T, Virkki R, Mertsola J (2000). Serum procalcitonin, C-reactive protein and interleukin-6 for distinguishing bacterial and viral pneumonia in children.. Pediatr Infect Dis J.

[47] Swischuk LE, Hayden CK (1986). Viral vs. bacterial pulmonary infections in children (is roentgenographic differentiation possible?).. Pediatr Radiol.

[48] Virkki R, Juven T, Rikalainen H, Svedstrom E, Mertsola J (2002). Differentiation of bacterial and viral pneumonia in children.. Thorax.

[49] Bossuyt PM, Reitsma JB, Bruns DE, for the STARD Group (2003). Towards complete and accurate reporting of studies of diagnostic accuracy: The STARD Initiative.. Ann Intern Med.

[50] Jones J, Hunter D (1995). Consensus methods for medical and health services research.. BMJ.

[51] Alessandrini EA, Alpern ER, Chamberlain JM, Shea JA, Gorelick MH (2010). A new diagnosis grouping system for child emergency department visits.. Acad Emerg Med.

