# Supplementary material for: A Systematic Review on the Diagnosis of Pediatric Bacterial Pneumonia: When Gold Is Bronze
Source: PLoS One. 2010 Aug 6;5(8):e11989. doi: 10.1371/journal.pone.0011989 (PMC2917358; doi:10.1371/journal.pone.0011989)
Supplement: Table S1 — Study Characteristics. (0.07 MB PDF) [file pone.0011989.s001.pdf]

Table S1: Childhood Bacterial Pneumonia: Study Characteristics

**Author; Year; Country; Funding; QUADAS score**	**Study Design (test administration); Study Setting; Recruitment Procedure**	**Gold Standard; Category Name: Description of Standard**	**Index Test; Category Name: Description of Test**
Bettenay; 1988; Australia; NR; 9	Retrospective cohort (index test followed by gold standard); Children's hospital (inpatients and outpatients); NR	Microbiology: Full blood picture and CRP; blood cultures including anaerobic and aerobic cultures, nasopharyngeal aspirates for immunofluorescent staining and cultures for viruses, m.pneu, c. trachomatis, bordetella pertussis, urine at admission and for the next two days for H. influenzae, and strep. Pneu. Antigens by LPAT, paired serum for Respiratory Syncytial Virus.	Symptoms & Hematologic: Clinical diagnosis based on guidelines of Swischuk and Hayden to distinguish bacterial from viral pneumonia (duration of symptoms; fever; total WBC; response to antibiotics).; Chest Radiograph: Reviewed by two radiologists using schema of Swischuk and Hayden to distinguish bacterial from viral pneumonia.
Blackmore; 1995; Australia; NR; 11	Prospective cohort (index test followed by gold standard); Hospital; NR	Hematologic: Particle-agglutination test used to detect antibodies to M. pneumonia. A stable titer of ≤1:80 over 10 days regarded as negative; a single titer of ≤1:80 indeterminate; and a single titer of ≥1:160 or a three-fold rise in titer as positive.	Immunologic: Swabs were taken for M. Pneumonia, PCR testing. The detection limit was ≥10-20 Colony Forming Units per reaction-tube for the MPN set, and 30-40 Colony Forming Units for the P1 set of primers.
Castriota-Scanderberg; 1995; Italy; NR; 10	Retrospective cohort (index test followed by gold standard); Hospital; NR	Microbiologic: Tested twice for serum antimycoplasm antibodies	Chest Radiograph: Bilateral parallel; Lobular or segmented with or without pleural consolidation; Unilateral node infiltrate; Peripheral and diffuse infiltrate
Don; 2007; Italy; NR; 14	Prospective cohort (gold standard followed by index test); Pediatric department; Consecutive	Chest Radiograph: Three experienced radiologists, unaware of the aetiology and the interpretation of the initial radiographs, individually and independently evaluated all the radiographs and the final radiological diagnosis was considered when at least 2 agreed.	Immunologic: Serum PCT samples obtained on admission, measured by immunoluminometric assay with 2 monoclonal antibodies
Esposito; 2002; Italy; Industry; 12	Prospective cohort (index test followed by gold standard); Pediatric department; NR	Hematologic & Immunologic: acute pneumococcal infection was diagnosed if the patient showed a ≥3-fold increase in the concentrations of type-specific anticapsular IgG ≥1 of the 9 tested serotypes in a comparison of paired serum samples. Acute M. and/or C. pneumoniae infection was diagnosed by the recommended serological means of determining the prevalence of infection in epidemiological studies.	Chest Radiograph: (hyperinflation, peribronchial wall thickening, perihilar linear opacities, reticulonodular infiltrate, segmental or lobar consolidation, bilateral consolidations); Signs & Symptoms: cough, tachypnea, fever, rales, wheezing

Esteban; 1995; Spain; NR; 10	Prospective cohort (index test followed by gold standard); Hospital; Consecutively admitted over two-year period	Signs & Symptoms / Chest Radiograph: Clinical diagnosis of URTI, laryngitis, bronchitis, bronchiolitis, or pneumonia	Microbiologic: serology by CF test carried out in 42 patients older than 6 months.; Virologic: nasopharyngeal aspirates collected for culture on cell monolayers in 87 patients.
Gambert; 1993; France; NR; 10	Retrospective cohort (gold standard followed by index test) ; Hospital; Arbitrary / Random	Serology: two techniques were used: CF and particle agglutination.	Signs and Symptoms included: 1) General Malaise; 2) Abdominal symptoms: pain, diarrhea, vomiting; 3) Postnasal drip; 4) Otitis Media; 5) Sinusitis; 6) Tonsilitis; 7) Cutaneous symptoms; 8) Cough; 9) Dyspnea (shortness of breath); 10) Thoracic pain; 11) Auscultory signs
Gendrel; 2002; France; NR; 9	Prospective cohort (gold standard followed by index test); Emergency department; NR	Microbiologic: bacterial probable when more than 25 polymorphonuclear cells and fewer than 10 squamous epithelial cells/mL were detected in Gram stains and cultures containing a single or a predominant microorganism with more than 10^6 cfu/mL present in the samples	Immunologic: Blood samples collected in vials containing EDTA. Leukocyte count measured automatically by hospital lab and serum CRP determined by nephelometry. PCT determined by frozen samples by an immunoluminometric assay.
Hardy; 2003; USA; NR; 9	Prospective cohort (index test followed by gold standard); Children's hospital; Consecutive	Immunologic: children diagnosed with M. pneumoniae by IgM and/or IgG ELISA with acute and/or convalescent serum samples.	Microbiologic: nasopharyngeal and oropharyngeal swabs were performed on all subjects and saved at -80°C
Jimenez; 1997; Spain; NR; 9	Prospective cohort (gold standard followed by index test); Pediatrics ward of hospital; Consecutively hospitalized over 18 months for hypothermia, vomiting, dehydration, or difficulty breathing	Signs & Symptoms: fever, cough, vomiting, skin retraction, tachypnea, wheezing, crackles, rash, running nose; Chest Radiograph: local condensation, diffuse condensation, trappings, hilar reinforcement; Hematologic: segmented, lymphocytes, monocytes	Microbiologic: Serology with titer ≥ 1/64 by CF
Liu; 2007; Taiwan; NR; 11	Prospective cohort (index test followed by gold standard) ; Pediatric Department; NR	Immunologic: Commercially available microparticle agglutination assay was used for determination of antibody titers (IgG and IgM) against M. pneumoniae. ELISA was used for the semiquantitative detection of specific IgM antibodies to M. pneumoniae in human serum.	Microbiologic: PCR was performed using a M. pneumoniae attachment protein P1 Primer Set Kit.

Mayoral; 2005; Argentina; NR; 10	Prospective cohort (gold standard followed by index test); Hospital; NR	Microbiologic, Group A: serum samples obtained over the course of the illness when Streptococcus pneumoniae was isolated from blood culture; Chest Radiograph, Group B: chest radiograph showed segmental, lobar or multilobar consolidation in all cases	Microbiologic: Nested-PCR: two rounds of PCR; analyzed using electrophoresis on 2% agarose/1X TAE buffer gels then observed under UV illumination.; Group A & B: Serum (100 µl) was diluted with an equal volume of 0.1 M HCl-Tris buffer pH 8.0. The mixture was incubated at 100 ^{0}C for 14 minutes, centrifuges at 10000g during 5 min and the supernatant was stored at 4 ^{0}C until PCR assay.
Moulin; 2001; France; Other (hospital); 12	Prospective cohort (gold standard followed by index test); Emergency department; NR	Hematologic: Blood samples were collected in EDTA by venepuncture, taken for serological testing, tests were performed on admission and repeated 2-3 weeks later. Sputum samples (or pharyngeal aspiration in those below the age of 5 years) were taken from all children to test for bacteria and viruses according to usual procedures.	Hematologic and Immunologic: In total there were 7 index tests performed all using plasma taken from blood samples. Plasma samples remaining after C-reactive protein and WBC counts were frozen for later determination of PCT. Leukocyte count was determined automatically by hospital laboratory and serum CRP was determined by nephelometry. Interleukin-6 (IL-6) was determined in the plasma that remained after PCT assay. PCT was determined by an immunoluminometric assay and IL-6 was determined by ELISA.
Nadal; 1999; Switzerland; NR; 10	Prospective cohort (not reported); Other (hospital); During a 12-month period	Immunologic: seroconversion to M. pneumoniae in paired sera as determined by CF or detection of specific IgM and IgG by ELISA	Microbiologic: nasopharyngeal secretions and pharyngeal swabs PCR was employed
Nagayama; 1988; Japan; NR; 10	Prospective cohort (gold standard followed by index test); Pediatric ward of hospital; Arbitrary	Microbiology: Isolation of M. pneumoniae obtained from throat swab and identification was confirmed by growth inhibition with specific rabbit antiserum to M. pneumoniae.	Immunologic: 1) Indirect hemagglutination test used to detect antibodies to M. pnuemoniae using standard microtechniques (Serodia Myco®); 2) CF-test used to detect antibodies to M. pnuemoniae using standard microtechniques (MP Antigen Eiken®)
Nunes; 2004; Brazil; Government; 13	Prospective cohort (index test followed by gold standard); NR ; NR	Reference diagnosis comprising the presence of following abnormalities in all 'Cases'; Signs & Symptoms – fever ≥ 38^{0}C, respiratory rate > 40 respirations/min plus chest indrawing according to World Health Organization criteria; Hematologic/Immunologic – WBC count ≥15,000/mm^3 with polymorphonuclear leukocytes > 70% and band count ≥ 500/mm^3 and CRP ≥40 mg/l; Radiological – chest x-ray films presenting a Khamapirad and Glezen score ≥ 2, indicating a high probability of diagnosis of bacterial pneumonia.	Microbiologic: LPAT was performed in concentrated urine specimens that were obtained through spontaneous voiding in sterile plastic flasks, and immediately taken to the laboratory. Samples were submitted to a physicochemical concentration by adding a solution of ethanol-acetone in a 1:1 ration in a volume equal to the urine samples. Mixture was cooled, centrifuged, and impurities were removed. Results were read within 2 minutes of reagent addition with the naked eye aided by artificial light.

Prat; 2003; Spain; NR; 10	Prospective cohort (gold standard followed by index test); Pediatric emergency department; NR	Microbiologic: blood cultures; nasopharyngeal aspirates for viral studies; serum for antibody assays; urine for antigen detection; and pleural fluid cultures when pneumonia was associated with pleural effusion. Those with pneumococcal pneumonia (n=31) had streptococcus pneumoniae isolated in blood cultures (5 patients) and also in pleural fluid (2 patients). Remainder diagnosed with detection of pneumococcal capsular polysaccharide antigen in urine samples by counterimmunoelectrophoresis. Those in the atypical pneumonia group (n=20) included 18 diagnosed with mycoplasma pneumoniae by agglutinating gelatin particles sensitized with M. pneumoniae cell components and 2 with Chlamydia pneumoniae by microimmunofluorescence.	Immunologic: 1) PCT: serum samples for PCT measurement were stored at -20°C. A cutoff point of 2 ng/ml was used; 2) CRP: a serum sample was collected on admission and measured during routine diagnostic evaluation. A cutoff point of 65 mg/l was utilized.
Requejo; 2007; Brazil; NR; 8	Prospective cohort (gold standard followed by index test); NR; NR	Microbiologic: Bacterial cultures of pleural fluid samples	Immunologic & Microbiologic: Counterimmunoelectrophoresis, LPAT, dot-enzyme-linked immunosorbent assay (Dot-ELISA). A total of nine tests were performed counterimmunoelectrophoresis, latex agglutination, and Dot-ELISA was each performed on pleural fluid, serum, and urine samples.
Saha; 2006; Bangladesh; NR; 9	Prospective (gold standard followed by index test); Children's hospital; NR	Microbiologic: Blood cultures	Microbiologic: LPAT was performed using urine sample
Swischuk; 1986; USA; NR; 11	Prospective cohort (index test followed by gold standard); Hospital; Consecutive	Signs & Symptoms: 1) duration of illness and degree of presenting fever (sudden onset and high fevers 103 or over considered bacterial); 2) response to antibiotic therapy (brisk and prompt response considered consistent with bacterial infection, failure of such a response was believed more consistent with viral or m.pneu infection; Hematologic: 3) total and differential WBC count (lymphocytosis considered viral, high neutrophil counts with increased bands favored bacterial); 4) positive cold-agglutinants for mycoplasma pneumoniae infection; Two or more of the above parameters needed to be positive for a diagnosis of bacterial pneumonia.	Chest Radiograph: Roentgenographic differentiation: 1) parahilar peribronchial infiltrate with or without atelectasis (viral or m. pneu); 2) lobar consolidation, homogenous or fluffy (bacterial or m. pneu); 3) reticulonodular infiltrate restricted to one lobe (m. pneu); 4) diffuse bilateral fluffy infiltrates extending in to the periphery of the lungs (bacterial); 5) parihilar peribronchial infiltrates with peripheral consolidation (viral with superimposed bacterial); 6) indeterminate infiltrates

Toikka; 2000; Finland; Foundation; 13	Prospective cohort (index test followed by gold standard); University hospital; NR	Immunologic and Virologic: The diagnosis of bacterial pneumonia was based on high single values or a significant rise in antibody titers between acute and convalescent sera, detection of immune complexes in the serum or detection of nucleic acid or detection of live M. pneumoniae in nasopharyngeal aspirates.	Immunologic: In total there are 6 index tests all using serum samples taken on admission or on the first morning. CRP was analyzed using an immunoturbidometric method with a Hitachi 717 apparatus: 1) CRP cutoff of 80 mg/l; 2) CRP cutoff of 150 mg/l. PCT was measured by immunoluminometric assay with the LUMItest PCT kit: 3) PCT cutoff 2.0 ng/ml; 4) PCT cutoff 7.0 ng/ml. IL-6 concentrations were quantified by chemiluminescent enzyme immunoassay: 5) IL-6 cutoff 40 pg/ml; 6) IL-6 cutoff 100 pg/ml.
Tsai; 2004; Taiwan; NR; 10	Prospective cohort (gold standard followed by index test); Children's hospital; Consecutive	Microbiologic: Antigen detection and serologic methods. Antigen detection in sputum performed by IFA, antibody response measured by MIF. Criteria were either positive sputum antigen, 4-fold or higher rise in IgG titers, single positive IgM result in serum or an IgG titer ≥1: 640	Signs & Symptoms: clinical signs and symptoms at admission; Hematologic: leukocyte and platelet counts; Immunologic: CRP
Tzeng; 2005; Taiwan; NR; 10	Retrospective cohort (gold standard followed by index test); Veterans hospital (tertiary care); NR	Microbiologic: For pediatric patients: a diagnosis of S. pneumonia required a positive S. pneumonia culture result from blood or pleural effusion.	Immunologic: Immunochromatographic Membrane Assay: Unconcentrated urine, tested with rabbit anti S. pneumonia-conjugated antibody to bind any soluble pneumococcal antigen, and resulting antigen-antibody complexes captured by immobilized anti-sP antibodies, forming the sample line
Virkki; 2002; Finland;Academic and Foundation; 10	Prospective cohort (gold standard followed by index test); Hospital; Consecutive	Chest radiograph: posterior and lateral chest radiographs obtained on admission reviewed retrospectively and separately by 3 pediatric radiologists, classified findings as alveolar and/or interstitial pneumonic changes, hyperaeration, hilar enlargement, atelectasis, pleural fluid, location in one lung or both lungs.	Hematologic: WBC counts and erythrocyte sedimentation rate; Immunologic: serum CRP levels
Vuori-Holopainen; 2002; Finland; Industry; 11	Prospective cohort (index test followed by gold standard); Children's hospital; NR	Hematologic: blood culture of pneumococcal isolates were serotyped with the use of counterimmunolectrophoresis and the LPAT; Microbiologic: PCR—nucleic acid isolation methods selected to allow recovery of both DNA and RNA from the samples, isolated with a QIAamp tissue kit; Immunologic: DNA-RNA hybridization performed for pneumococcus (RNA-hyb)	Microbiologic: lung aspirate sample sent for standard bacteriological testing; cultured in thioglycolate growth medium using acridine orange staining. If bacteria were detected, Gram staining was also performed. Aspiration fluid specimen was considered representative if leulocytes were seen by microscopic examination.

CF, complement fixation; CRP, C-reactive protein; EDTA, ethylenediaminetetraacetic acid; ELISA, enzyme-linked immunosorbent assay; ESR, erythrocyte sedimentation rate; IL-6, Interleukin-6; LPAT, latex agglutination particle test; NR, not reported; PCR, polymerase chain reaction; PCT, procalcitonin; QUADAS, quality assessment of studies of diagnostic accuracy included in systematic reviews; URTI, upper respiratory tract infection; WBC, white blood cell
