# Supplementary material for: A Systematic Review on the Diagnosis of Pediatric Bacterial Pneumonia: When Gold Is Bronze
Source: PLoS One. 2010 Aug 6;5(8):e11989. doi: 10.1371/journal.pone.0011989 (PMC2917358; doi:10.1371/journal.pone.0011989)
Supplement: Table S2 — Participant Characteristics. (0.06 MB PDF) [file pone.0011989.s002.pdf]

**Table S2: Childhood Bacterial Pneumonia: Participant Characteristics**

| Author; Year | Total Number of Participants; Enrolled/Analyzed | Age (Mean ± SD) [Median; Range]; Males: Number (%) | Definition of Pneumonia | Inclusion Criteria |
|---|---|---|---|---|
| Bettenay; 1988 | 107 patients reviewed; 58 patients with complete information; formed study group | NR ; NR | Atypical (Mycoplasma) and Typical (Streptococcus) | Previously healthy children older than 100 days with strong clinical evidence of pneumonia. |
| Blackmore; 1995 | 203 patients admitted; 99 patients entered in study; 46 were children | Under 16 yrs; Further Information not given for children as a group | Atypical (Mycoplasma) | Patients admitted to hospital and investigated serologically for M. pneumoniae infection during the study period. |
| Castriota-Scanderberg; 1995 | 120 charts reviewed for x-ray and clinical data; 76 children /adolescents charts included; ; M. pneumonia = 38; Viral pneumonia = 22; Bacterial pneumonia = 13; Mixed etiology = 3 | Median: 10.4; SD 1.3; Range: 4.2-16.4 yrs Male: 42 (55.2%) | Atypical (Mycoplasma) | Children and adolescents with a radiographic diagnosis of pneumonia (showing pulmonary infiltration). |
| Don; 2007 | 125 evaluated (25 excluded: appropriate serum samples or chest radiograph not available; not diagnosed with pneumonia); 100 included | Median: 3.7 yrs; Male: 49 (49%) | Atypical (Mycoplasma; Chlamydia) and Typical (Streptococcus) | Previously healthy children; clinical suspicion of pneumonia. |
| Esposito; 2002 | Enrolled = 196; Analyzed = 110 | Mean ± SD: 3.707 ± 0.870 yrs; Male: 50.5% | Typical (Streptococcus) and atypical bacteria alone and combined | Previously healthy children 2-5 yrs of age with signs; symptoms; and chest radiograph findings consistent with community acquired pneumonia.; No concomitant disease; nosocomially acquired infection or antibiotics in past 48 hours. |
| Esteban; 1995 | 87 patients enrolled; ; upper RTI = 19; Laryngitis = 3; Bronchitis = 18; Bronchiolitis = 18; Pneumonia = 29 | Mean: 2 yrs; Range: 1 month to 14 yrs; Males: 64% | Atypical (Mycoplasma) | Diagnosis of acute RTI with no other baseline conditions; diagnosis based on signs and symptoms at entry and chest radiograph. |
| Gambert; 1993 | M. pneumonia = 19; Other types of pneumonia = 21; NR; NR | Median: 6 yrs; Range: 3-12 yrs; Males: 8 (42.1%) those with M. pneumonia | Atypical (Mycoplasma) | Children with a diagnosis of Mycoplasma pneumonia; as diagnosed with the serological criteria. |

| | | | | |
|---|---|---|---|---|
| Gendrel; 2002 | Enrolled = 88; Analyzed = 72 | Range: 2 months – 13 yrs; NR | Bacterial pneumonia | Children free of chronic disease; and who had not received antibiotics 2 weeks prior to admission.; All patients had fever over 38.5$^{o}$C; and chest x-ray showing clear signs of pneumonia with alveolar infiltration. |
| Hardy; 2003 | Enrolled = 154; Underwent both tests = 62 | NR; NR | Atypical (Mycoplasma) | Immunocompetent children with radiographically-confirmed community acquired pneumonia. |
| Jimenez; 1997 | 56 inpatients enrolled and diagnosed with pneumonia; Viral = 20; Bacterial = 16; Unknown = 20 | Mean: 3 yrs; Range: 2 months – 14 yrs; Male: 54%; NR | Atypical (Mycoplasma; Chlamydia) and Typical (Streptococcus; H influenzae; M. tuberculosis) | Clinical history. |
| Liu; 2007 | Available for inclusion = 307; Patients included = 256; Serological tests performed and included = 140 | Range: 3 months - 16 yrs (based on 307 available for inclusion); Males: 130 (50.8%) (based on 256 included in study) | Atypical (Mycoplasma) | Hospitalized children with RTI. |
| Mayoral; 2005 | Group A = 24; Group B = 75 | Range: 3 months – 5 yrs; None reported | Typical (Streptococcus) | Pediatric patients 3 months to 5 yrs.; Group A: children with definitive diagnosis of pneumococcal pneumonia confirmed by blood-culture.; Group B: children with clinical and radiological evidence of bacterial pneumonia. |
| Moulin; 2001 | Admitted for community acquired pneumonia = 88; Had no pathogen identified and excluded = 16; Analyzed for bacterial or viral pathogen = 72 | S. Pneumonia (n=10): Mean 1.9 yrs; Range 0.4-5 yrs; Bacterial Pneumonia (n=15): Mean 3.9 yrs; Range 0.5-14 yrs; Viral Pneumonia (n=29): Mean 1.7 yrs; Range 0.5-7 yrs; Viral by Serology (n=8): Mean 1.3 yrs; Range 0.5-5 yrs; M. Pneumonia (n=10): Mean 6.2 yrs; Range 3-10 yrs | Atypical (Mycoplasma; Chlamydia) | Immunocompetent children without chronic disease; pulmonary or otherwise; and who had not received antibiotics in the 10 days before admission. |
| Nadal; 1999 | Enrolled = 168; Analyzed as paired sera available = 108 | Mean: 4.2 yrs; Range: 0.2-14.9 yrs; None reported | Atypical (Mycoplasma) | Radiologically confirmed community acquired pneumonia. |
| Nagayama; 1988 | Tested 1485 isolates for M. pneumoniae | Range: 0 – 14 yrs; None reported | Atypical (Mycoplasma) | Infants and children <=14 yrs of age with lower RTI manifestations were selected. |

| | | | | |
|---|---|---|---|---|
| Nunes; 2004 | Cases = 45; Controls = 62 | All Range: 2-12 yrs; Cases: Mean ± SD: 4.9 ± 2.9 yrs Males = 25 (56%) ; Controls: Mean ± SD: 5.6 ± 2.5 yrs Males = 27 (44%) | Detecting the capsular polysaccharide antigens of typical (Streptococcus; H influenzae) | Patients excluded if they had pleural effusion; pulmonary abscess; concomitant chronic pulmonary diseases or urinary tract infection; and clinical or radiological features suggesting viral and/or atypical pneumonia; such as interstitial and/or reticulonodular infiltrates. |
| Prat; 2003 | Clinically; radiologically; and microbiologically diagnosed with lower RTI = 85; Pneumococcal pneumonia = 31; Atypical pneumonia = 20; Viral pneumonia or bronchiolitis = 34; Control = 38 | Range between 6 months and 10 yrs; NR | Typical (Pneumococcal; Streptococcus) and Atypical (Mycoplasma; Chlamydia) | Children with clinical signs of lower RTI (fever; difficulty in breathing; or a pneumonic infiltrate in a chest radiograph). |
| Requejo; 2007 | Children included = 550 | Not stated; Not stated | Typical (Pneumococcal; Streptococcus) | Clinical diagnosis of bacterial pneumonia. |
| Saha; 2006 | Healthy children = 201; Pneumonia = 974; Meningitis = 127 | Mean: 9.5 months (pneumonia/ meningitis group | Haemophilus influenzae type b | Diagnosis of pneumonia was based on World Health Organization definition of acute lower RTI (respiratory rate of at least 50/min for children 2-12 months and 40/min for those aged 12-23 months; and chest indrawings. |
| Swischuk; 1986 | Enrolled = 110; Excluded due to conflicting concurrent infections and poor clinical documentation = 26; Analyzed = 84 | Range: 2 months – 15 yrs; “approximately equal” | Atypical (Mycoplasma) | Roentgenographic evidence of pulmonary infiltrates. |
| Toikka; 2000 | Initially enrolled = 254; Included and analyzed based on availability of serum samples = 126 | Median: 2.6 yrs; Range: 1 month to 17 yrs; Male: 52% | Community acquired pneumonia included: Atypical (Mycoplasma; Chlamydia) and Typical (Streptococcus; Moraxella Catarrhalis; H influenzae; S. pyogenes) | Availability of serum samples; signs and symptoms; chest x-rays; not immunocompromised.; Diagnosis of pneumonia based on simultaneous finding of fever and/or respiratory symptoms and infiltrates compatible with pneumonia on chest radiographs. |
| Tsai; 2004 | Enrolled with community acquired pneumonia = 209; Chlamydial infection = 26 | Range: 12 months -14 yrs 4 months ; Median: 3.5 yrs; Males: 14 (54%) | Atypical (Chlamydia) | Previously healthy children 3 months to 18 yrs admitted to hospital due to community acquired pneumonia. |
| Tzeng; 2005 | Adults and children enrolled = 1334; Children = 91 | Pts were <15 yrs of age.; No further demographic info provided | Typical (Streptococcus) | Met study criteria for RTI: Fever >38$^{o}$C; and one of the following conditions; cough; sore throat; otalgia; radiological signs of pulmonary involvement. |

| | | | | |
|---|---|---|---|---|
| Virkki; 2002 | Children enrolled = 296; Children entered in study = 254; Analyzed = 215 | 37 = < 1 year; 71 = 1-2 yrs; 84 = 2-5 yrs; 62 = ≥ 5 yrs; NR; No data | Atypical (Mycoplasma; Chlamydia) and Typical (Streptococcus; Moraxella Catarrhalis; H influenzae) | Children with community acquired pneumonia; based on simultaneous finding of an infiltrate on the chest x-ray and fever ≥ 37.5$^{o}$C and/or respiratory symptoms. |
| Vuori-Holopainen; 2002 | 47 asked to participate; 13 refused; Aspiration fluid sample obtained from 26 patients; analyses performed for 34 children. | Based on 34 children with pneumonia; Mean: 5.1 yrs (0.8-14.0 range); Males: 20 (59%) | Typical (Streptococcus) | Hospitalized children with pneumonia (respiratory symptoms and signs with a history of fever and consolidation in the chest radiograph at admission); Patients not immunocompromised; postoperative; did not have bleeding diathesis; were not treated in oncology or intensive care unit. |

NR, not reported; RTI, respiratory tract infection; SD, standard deviation; yrs, yrs
