# Supplementary material for: A Systematic Review on the Diagnosis of Pediatric Bacterial Pneumonia: When Gold Is Bronze
Source: PLoS One. 2010 Aug 6;5(8):e11989. doi: 10.1371/journal.pone.0011989 (PMC2917358; doi:10.1371/journal.pone.0011989)
Supplement: Table S3 — Criterion Validity for the Diagnosis of Bacterial Pneumonia. (0.06 MB PDF) [file pone.0011989.s003.pdf]

Table S3: Criterion Validity for the Diagnosis of Bacterial Pneumonia

Author; Year	Ref. Standard; / Index Test	True Positive	False Negative	False Positive	True Negative	Sensitivity; (95% CI)	Specificity; (95% CI)
Bettenay; 1988	Microbiology / Symptoms & Hematologic	3	8	14	33	27.3% (6.0 – 61.0%)§	70.2% (55.1 – 82.7%)§
	Microbiology / Chest Radiograph	6	2	14	24	75.0% (34.9 – 96.8%)§	63.2% (46.0 – 78.2%)§
Blackmore; 1995	Hematologic / MP-PCR (intermediate combined with negative titer)	22	1	3	20	95.7% (78.1 – 99.9%)§	87.0% (66.4 – 97.2%)§
Castriota-Scanderberg; 1995	Microbiologic / Bilateral parallel	10	28	19	19	26.3% (13.4 – 43.1%)§	50.0% (34.1 – 65.9%)*
	Microbiologic / Lobular or segmented with or without pleural consolidation	6	32;	18	20	15.8% (6.0 – 31.3%)§	52.6% (36.8 – 68.5%)*
	Microbiologic / Unilateral node infiltrate	22	16	0	38	57.9% (42.2 – 73.6%)*	100.0% (90.8 - 100%)§
	Microbiologic / Peripheral and difuse infiltrate	0	38	1	38	0% (0 – 9.3%)§	97.4% (86.5 – 99.9%)§
Don; 2007	<u>S. Pneumoniae</u>:; Radiograph / PCT>0.5 ng/ml	14	4	15	8	77.8% (52.4 – 93.6%)§	34.8% (16.4 – 57.3%)§
	<u>S. Pneumoniae</u>:; Radiograph / PCT>1.0 ng/ml	10	8	9	14	55.6% (30.8 – 78.5%)§	60.9% (38.5 – 80.3%)§
	<u>S. Pneumoniae</u>:; Radiograph / PCT>2.0 ng/ml	8	10	6	17	44.4% (21.5 – 69.2%)§	73.9% (51.6 – 89.8%)§
	<u>M & C. Pneumoniae</u>:; Radiograph / PCT>0.5 ng/ml	18	7	15	8	72.0% (50.6 – 87.9%)§	34.8% (16.4 – 57.3%)§
	<u>M & C. Pneumoniae</u>:; Radiograph / PCT>1.0 ng/ml	16	9	9	14	64.0% (42.5 – 82.0%)§	60.9% (38.5 – 80.3%)§
	<u>M & C. Pneumoniae</u>:; Radiograph / PCT>0.5 ng/ml	10	15	6	17	40.0% (21.1 – 61.3%)§	73.9% (51.6 – 89.8%)§
Esposito; 2002	Hematologic & Immunologic / Hyperinflation	13	97	11	75	11.8% (6.5 – 19.4%)§	87.2% (78.3 – 93.4%)§
	Hematologic & Immunologic / Peribronchial wall thickening	8	102	7	79	7.3% (3.2 – 13.8%)§	91.9% (84.0 – 96.7%)§
	Hematologic & Immunologic / Perihilar linear opacities	44	66	39	47	40.0% (30.8 – 49.2%)*	54.7% (44.1 – 65.2%)*
	Hematologic & Immunologic / Retinculonodular infiltrate	39	71	31	55	35.5% (26.5 – 44.4%)*	64.0% (53.8 – 74.1%)*
	Hematologic & Immunologic / Segmental or lobar consolidation	35	75	27	59	31.8% (23.1 – 40.5%)*	68.6% (58.8 – 78.4%)*
	Hematologic & Immunologic / Bilateral consolidations	13	97	10	76	11.8% (6.5 – 19.4%)§	88.4% (79.7 – 94.3%)§

	Hematologic & Immunologic / Pleural effusion	7	103	6	80	6.4% (2.6 – 12.7%)§	93.0% (85.4 – 97.4%)§
	Hematologic & Immunologic / Cough	75	35	60	26	68.2% (59.5 – 76.9%)*	30.2% (20.5 – 39.9%)*
	Hematologic & Immunologic / Tachypnea	27	83	21	65	24.6% (16.5 – 32.6%)*	75.6% (66.5 – 84.7%)*
	Hematologic & Immunologic / Fever ≥ 37.8	95	15	73	13	86.4% (80.0 – 92.8%)*	15.1% (8.3 – 24.5%)§
	Hematologic & Immunologic / Rales	101	9	79	7	91.8% (85.0 – 96.2%)§	8.1% (3.3 – 16.1%)§
	Hematologic & Immunologic / Wheezing	15	95	11	75	13.6% (7.3 – 20.1%)*	87.2% (78.3 – 93.4%)§
Esteban; 1995	Clinical / Microbiologic	16	5	9	12	76.2% (52.8 – 91.8%)§	57.1% (34.0 – 78.2%)§
	Clinical / Virologic	10	19	22	36	34.5% (17.9 – 54.3%)§	62.1% (49.6 – 74.6%)*
Gambert; 1993	Serology / General Malaise	12	7	15	6	63.2% (38.4 - 83.7%)§	28.6% (11.3 – 52.2%)§
	Serology/ Abdominal symptoms	9	10	9	12	47.4% (24.5 – 71.1%)§	57.1% (34.0 – 78.2%)§
	Serology/ Postnasal drip	15	4	12	9	79.0% (54.4 – 94.0%)§	42.9% (21.8 – 66.0%)§
	Serology / Otitis media	4	15	4	17	21.1% (6.1 – 45.6%)§	81.0% (58.1 – 94.6%)§
	Serology / Sinusitis	2	17	0	21	10.5% (1.3 – 33.1%)§	100% (83.9 – 100%)§
	Serology / Tonsilitis	2	17	3	18	10.5% (1.3 – 33.1%)§	85.7% (63.7 – 97.0%)§
	Serology /Cutaneous symptoms	5	14	3	18	26.3% (9.2 – 51.2%)§	85.7% (63.7 – 97.0%)§
	Serology / Cough	4	15	2	19	21.1% (6.1 – 45.6%)§	90.5% (69.6 – 98.8%)§
	Serology / Dyspnea	1	18	1	20	5.3% (0.1 – 26.0%)§	95.2% (76.2 – 99.9%)§
	Serology / Thoracic pain	2	17	2	19	10.5% (1.3 – 33.1%)§	90.5% (69.6 – 98.8%)§
	Serology / Auscultory signs	17	2	16	5	89.5% (66.9 – 98.7%)§	23.8% (8.2 – 47.2%)§
Gendrel; 2002	Microbiologic / PCT > 0.5 ug/L	41	2	10	19	95.4% (84.2 – 99.4%)§	65.5% (45.7 – 82.1%)§
	Microbiologic / PCT >1	37	6	4	25	86.1% (72.1 – 94.7%)§	86.2% (68.3 – 96.1%)§
	Microbiologic / PCT>2	27	16	1	28	62.8% (48.3 – 77.2%)*	96.6% (82.2 – 99.9%)§
	Microbiologic / CRP >20 mg/L	38	5	15	14	88.4% (74.9 – 96.1%)§	48.3% (29.5 – 67.5%)§
	Microbiologic / CRP >60	30	13	7	22	69.8% (53.9 – 82.8%)§	75.9% (56.5 – 89.7%)§
	Microbiologic / IL6 >100pg/mL	12	8	2	10	60.0% (36.1 – 80.9%)§	83.3% (51.6 – 97.9%)§
	Microbiologic / Leucocytes >15000	28	15	6	23	65.1% (49.1 – 79.0%)§	79.3% (60.2 – 92.0%)§
	Microbiologic / Inf-alpha <2 UI/mL	30	6	4	22	83.3% (67.2 – 93.6%)§	84.6% (65.1 – 95.6%)§
Hardy; 2003	ELISA / Np Op swabs	12	9	1	40	57.1% (34.0 – 78.2%)§	97.6% (87.1 – 99.9%)§
Jimenez; 1997	Fever / Microbiologic	14	37	2	3	27.5% (15.9 – 41.7%)§	60.0% (14.7 – 94.7%)§
	Cough / Microbiologic	14	39	2	1	26.4% (15.3 – 40.3%)§	33.3% (0.8 – 90.6%)§
	Vomiting / Microbiologic	8	28	8	12	22.2% (10.1 – 39.2%)§	60.0% (36.1 – 80.9%)§
	Skin Retraction / Microbiologic	4	16	12	24	20.0% (5.7 – 43.7%)§	66.7% (49.0 – 81.4%)§

	Tachypnea / Microbiologic	2	23	14	17	8.0% (1.0 – 26.0%)[§]	54.8% (36.0 – 72.7%)[§]
	Wheezing / Microbiologic	3	17	13	23	15.0% (3.2 – 37.9%)[§]	63.9% (46.2 – 79.2%)[§]
	Crackles / Microbiologic	11	35	5	5	23.9% (12.6 – 38.8%)[§]	50.0% (18.7 – 81.3%)[§]
	Rash / Microbiologic	0	5	16	35	0.0% (0.0 – 52.2%)[§]	68.6% (55.9 – 81.4%)*
	Running Nose / Microbiologic	6	16	10	24	27.3% (10.7 – 50.2%)[§]	70.6% (52.5 – 84.9%)[§]
	Local Condensation / Microbiologic	10	23	6	17	30.3% (15.6 – 48.7%)[§]	73.9% (51.6 – 89.8%)[§]
	Diffuse Condensation / Microbiologic	6	18	10	22	25.0% (9.8 – 46.7%)[§]	68.8% (50.0 – 83.9%)[§]
	Trappings / Microbiologic	1	6	15	34	14.3% (0.4 – 57.9%)[§]	69.4% (54.6 – 81.8%)[§]
	Hilar Reinforcement / Microbiologic	3	16	13	24	15.8% (3.4 – 39.6%)[§]	64.9% (47.5 – 79.8%)[§]
	Segmented / Microbiologic	11	23	5	17	32.4% (17.4 – 50.5%)[§]	77.3% (54.6 – 92.2%)[§]
	Lymphocytes / Microbiologic	4	12	12	28	25.0% (7.3 – 52.4%)[§]	70.0% (53.5 – 83.4%)[§]
	Monocytes / Microbiologic	1	3	15	37	25.0% (0.6 – 80.6%)[§]	71.2% (58.8 – 83.5%)*
Liu; 2007	Immunologic / PCR;	51	25	13	51	67.1% (56.6 – 77.7%)[*]	79.7% (67.8 – 88.7%)[§]
Mayoral; 2005	Group A / Nested PCR	20	4	0	20	83.3% (62.6 – 95.3%)[§]	100% (83.2 – 100%)[§]
	Group B / Nested PCR	53	22	0	20	70.7% (60.4 – 81.0%)*	100% (83.2 – 100%)[§]
Moulin; 2001	Serological / PCT > 0.5 µg/l	41	2	10	19	95.4% (84.2 – 99.4%)[§]	65.5% (45.7 – 82.1%)[§]
	Serological / PCT > 1.0 µg/l	37	6	4	25	86.1% (72.1 – 94.7%)[§]	86.2% (68.3 – 96.1%)[§]
	Serological / PCT > 2.0 µg/l	27	16	1	28	62.8% (48.3 – 77.2%)[*]	96.6% (82.2 – 99.9%)[§]
	Serological / CRP > 20 mg/l	38	5	15	14	88.4% (74.9 – 96.1%)[§]	48.3% (29.5 – 67.5%)[§]
	Serological / CRP > 60 mg/l	30	13	7	22	69.8% (53.9 – 82.8%)[§]	75.9% (56.5 – 89.7%)[§]
	Serological / IL-6 > 100 pg/ml	12	8	2	10	60.0% (36.1 – 80.9%)[§]	83.3% (51.6 – 97.9%)[§]
	Serological / WBC >15;000 ($x10^6$/l)	28	15	6	23	65.1% (49.1 – 79.0%)[§]	79.3% (60.3 – 92.0%)[§]
Nadal; 1999	CF / PCR	8	14	3	83	36.4% (17.2 – 59.3%)[§]	96.5% (90.1 – 99.3%)[§]
	ELISA / PCR	7	6	4	91	54.9% (25.1 – 80.8%)[§]	95.8% (89.6 – 98.8) [§]
Nagayama; 1988	Microbiology / IHA <40	85	135	18	96	38.6% (32.2 – 45.1%)[*]	84.2% (77.5 – 90.9%)[*]
	Microbiology / IHA ≥640	83	137	68	46	37.7% (31.3 – 44.1%)[*]	40.4% (31.3 – 49.4%)[*]
	Microbiology / CF titer <4	38	91	15	66	29.5% (21.6 – 37.3%)[*]	81.5% (73.0 – 89.9%)[*]
	Microbiology / CF titer ≥64	49	80	45	36	38.0% (29.6 – 46.4%)[*]	44.4% (33.6 – 55.3%)[*]
Nunes; 2004	Reference Diagnosis / LPAT	34	10	6	56	77.3% (62.2 – 88.5%)[§]	90.3% (80.1 - 96.4%)[§]

Prat; 2003	1) Microbiologic / PCT at cutoff of 2 ng/ml	35	16	7	27	68.6% (56.0 – 81.4%)*	79.4% (62.1 – 91.3%)§
	2) Microbiologic / CRP at cutoff of 65 mg/l	40	11	11	23	78.4% (64.7 – 88.7%)§	67.7% (49.5 – 82.6%)§
Requejo; 2007	Microbiologic / CIE (pleural fluid)	146	0	52	352	100% (97.5 - 100%)§	87.1% (83.9 – 90.4%)*
	Microbiologic / LA (pleural fluid)	146	0	130	274	100% (97.5 - 100%)§	67.8% (63.3 – 72.4%)*
	Microbiologic / ELISA (pleural fluid)	146	0	204	200	100% (97.5 - 100%)§	49.5% (44.6 – 54.4%)*
	Microbiologic / CIE (serum)	133	13	0	404	91.1% (85.3 – 95.2%)§	100% (99.1 - 100%)§
	Microbiologic / LA (serum)	146	0	64	340	100% (97.5 - 100%)§	84.2% (80.6 – 87.7%)*
	Microbiologic / ELISA (serum)	146	0	176	228	100% (97.5 - 100%)§	56.4% (51.6 – 61.3%)*
	Microbiologic / CIE (urine)	146	0	57	347	100% (97.5 - 100%)§	85.9% (82.5 – 89.3%)*
	Microbiologic / LA (urine)	146	0	114	290	100% (97.5 - 100%)§	71.8% (67.4 – 76.2%)*
	Microbiologic / ELISA (urine)	146	0	173	231	100% (97.5 - 100%)§	57.2% (52.4 – 62.0%)*
Saha; 2006	Blood Culture / LAT	10	0	2	117	100% (69.2 - 100%)§	98.3% (94.1 – 99.8%)§
Swischuk; 1986	Clinical diagnosis / Chest radiograph	16	7	2	49	69.6% (47.1 – 86.8%)§	96.1% (86.5 – 99.5%)§
Toikka ; 2000	Immunologic / CRP cutoff 80 mg/l	40	28	13	27	58.8% (47.1 – 70.5%)*	67.5% (50.9 – 81.4%)§
	Immunologic / CRP cutoff of 150 mg/l	21	47	5	35	30.9% (19.9 – 41.9%)*	87.5% (73.2 – 95.8%)§
	Immunologic / PCT cutoff 2.0 ng/ml	34	34	8	32	50.0% (38.1 – 61.9%)*	80.0% (64.4 – 91.0%)§
	Immunologic / PCT cutoff 7.0 ng/ml	13	55	1	39	19.1% (10.6 – 30.5%)§	97.5% (86.8 – 99.9%)§
	Immunologic / IL-6 cutoff 40 pg/ml	23	45	7	33	33.8% (22.6 – 45.1%)*	82.5% (67.2 – 92.7%)§
	Immunologic / IL-6 cutoff 100 pg/ml	12	56	1	39	17.7% (9.5 – 28.8%)§	97.5% (86.8 – 99.9%)§
Tsai; 2004	Microbiologic / Fever	5	0	0	21	100% (47.8 - 100%)§	100% (83.9 - 100%)§
	Microbiologic / Cough	5	0	0	21	100% (47.8 - 100%)§	100% (83.9 - 100%)§
	Microbiologic / Rhinorrhea	3	2	13	8	60.0% (14.7 – 94.7%)§	38.1% (18.1 – 61.6%)§
	Microbiologic / Tachypnea	0	5	4	17	0.0% (0.0 – 52.2%)§	81.0% (58.1 – 94.6%)§
	Microbiologic / Wheeze	0	5	2	19	0.0% (0.0 – 52.2%)§	90.5% (69.6 – 98.8%)§
	Microbiologic / Rales	3	2	12	9	60.0% (14.7 – 94.7%)§	42.9% (21.8 – 66.0%)§
	Microbiologic / Rhonchi	1	4	6	15	20.0% (0.5 – 71.6%)§	71.4% (47.8 – 88.7%)§
	Microbiologic / Vomiting	1	4	3	18	20.0% (0.5 – 71.6%)§	85.7% (63.7 – 97.0%)§
	Microbiologic / Abdominal pain	0	5	1	20	0.0% (0.0 – 52.2%)§	95.2% (76.2 – 99.9%)§
	Microbiologic / Leukocyte count (/mm^{3}) <5000	1	4	3	18	20.0% (0.5 – 71.6%)§	85.7% (63.7 – 97.0%)§
	Microbiologic / Leukocyte count (/mm^{3}) 5000-15;000	3	2	11	10	60.0% (14.7 – 94.7%)§	47.6% (25.7 – 70.2%)§

	Microbiologic / Leukocyte count (/mm$^{3)}$ >15;000	1	4	7	14	20.0% (0.5 – 71.6%)§	66.7% (43.0 – 85.4%)§
	Microbiologic / Platelet count (10^{3}/mm$^{3)}$ ≥100	0	5	20	1	0.0% (0.0 – 52.2%)§	4.8% (0.1 – 23.8%)§
	Microbiologic / CRP (mg/L) <10	2	3	1	20	40.0% (5.3 – 85.3%)§	95.2% (76.2 – 99.9%)§
	Microbiologic / CRP (mg/L) 10-40	0	5	5	16	0.0% (0.0 – 52.2%)§	76.2% (52.8 – 91.8%)§
	Microbiologic / CRP (mg/L)>40	3	2	15	6	60.0% (14.7 – 94.7%)§	28.6% (11.3 – 52.2%)§
Tzeng; 2005	Microbiologic / ICT	10	1	25	55	90.9% (58.7 - 99.8%)§	68.8% (58.6 – 78.9%)*
Virkki; 2002	Radiograph / WBC >15.0 x 10^{9}/l	64	70	38	43	47.8% (39.3 – 56.2%)*	53.1% (42.2 – 64.0%)*
	Radiograph / ESR >30 mm/h	88	46	49	32	65.7% (57.6 – 73.7%)*	39.5% (28.9 – 50.2%)*
	Radiograph / CRP <20 mg/l	30	104	27	54	22.4% (15.3 – 29.5%)*	66.7% (56.4 – 76.9%)*
	Radiograph / CRP >40 mg/l	89	45	38	43	66.4% (58.4 – 74.4%)*	53.1% (42.2 – 64.0%)*
	Radiograph / CRP >80 mg/l	70	64	23	58	52.2% (43.8 – 60.7%)*	71.6% (61.8 – 81.4%)*
	Radiograph / CRP >120 mg/l	48	86	12	69	35.8% (27.7 – 43.9%)*	85.2% (75.6 – 92.1%)§
Vuori-Holopainen; 2002	Blood culture / Lung aspirate	1	9	1	9	10.0% (0.3 – 44.5%)§	90.0% (55.6 – 99.8%)§
	PCR / Lung aspirate	8	1	8	2	88.9% (51.8 – 99.7%)§	20.0% (2.5 – 55.6%)§
	RNA-hyb / Lung aspirate	10	0	0	8	100.0% (69.2 – 100%)§	100.0% (63.1 - 100%)§

*normal approximation used to calculate 95% confidence intervals; §binomial exact method used to calculate 95% confidence interval
