# Supplementary material for: A Systematic Review on the Diagnosis of Pediatric Bacterial Pneumonia: When Gold Is Bronze
Source: PLoS One. 2010 Aug 6;5(8):e11989. doi: 10.1371/journal.pone.0011989 (PMC2917358; doi:10.1371/journal.pone.0011989)
Supplement: Table S4 — Quality Assessment of Studies of Diagnostic Accuracy included in Systematic Reviews (QUADAS). (0.01 MB PDF) [file pone.0011989.s004.pdf]

Table S4: Childhood Bacterial Pneumonia: Quality Assessment of Studies of Diagnostic Accuracy included in Systematic Reviews (QUADAS)

Author; Year	QUADAS Question (see legend for each question)														Total
	1	2	3	4	5	6	7	8	9	10	11	12	13	14	
Bettenay; 1988	Unclear	Unclear	Yes	Unclear	Yes	Yes	Yes	Yes	Yes	Unclear	Unclear	Yes	Yes	Yes	9
Blackmore; 1995	Unclear	Yes	Yes	Unclear	Yes	Yes	Yes	Yes	Yes	Unclear	Yes	Yes	Yes	Yes	11
Castriota-Scanderberg; 1995	Unclear	Yes	Yes	Unclear	Yes	Yes	Yes	Yes	Yes	Unclear	Unclear	Yes	Yes	Yes	10
Don; 2007	Yes	Yes	Yes	Yes	Yes	Yes	Yes	Yes	Yes	Yes	Yes	Yes	Yes	Yes	14
Esposito; 2002	Unclear	Yes	Yes	Unclear	Yes	Yes	Yes	Yes	Yes	Yes	Yes	Yes	Yes	Yes	12
Esteban; 1995	Yes	Yes	Yes	Unclear	Yes	Yes	Yes	Yes	Yes	Unclear	Unclear	Yes	Unclear	Yes	10
Gambert; 1993	Yes	Yes	Yes	Unclear	Yes	Yes	Yes	Yes	Yes	Unclear	Unclear	Yes	Unclear	Yes	10
Gendrel; 2002	Unclear	Yes	Yes	Yes	Yes	Yes	Yes	Yes	Yes	Unclear	Unclear	Yes	Unclear	Unclear	9
Hardy; 2003	Unclear	Yes	Yes	Unclear	Yes	Yes	Yes	Yes	Yes	Unclear	Unclear	Yes	Unclear	Yes	9
Jimenez; 1997	Yes	Unclear	Yes	Unclear	Yes	Yes	Yes	Yes	Yes	Unclear	Unclear	Yes	Unclear	Yes	9
Liu; 2007	Unclear	Yes	Yes	Yes	Yes	Yes	Yes	Yes	Yes	Unclear	Unclear	Yes	Yes	Yes	11
Mayoral; 2005	Unclear	Yes	Yes	Yes	Yes	Yes	Yes	Yes	Yes	Unclear	Unclear	Yes	Unclear	Yes	10
Moulin; 2001	Unclear	Yes	Yes	Yes	Yes	Yes	Yes	Yes	Yes	Unclear	Yes	Yes	Yes	Yes	12
Nadal; 1999	Unclear	Yes	Yes	Yes	Yes	Yes	Yes	Yes	Unclear	Unclear	Unclear	Yes	Yes	Yes	10
Nagayama; 1988	Unclear	Yes	Yes	Unclear	Yes	Yes	Yes	Yes	Yes	Unclear	Unclear	Yes	Yes	Yes	10
Nunes; 2004	Unclear	Yes	Yes	Yes	Yes	Yes	Yes	Yes	Yes	Yes	Yes	Yes	Yes	Yes	13
Prat; 2003	Unclear	Yes	Yes	Yes	Yes	Yes	Yes	Yes	Yes	Unclear	Unclear	Yes	Unclear	Yes	10
Requejo; 2007	Unclear	Unclear	Yes	Unclear	Yes	Yes	Yes	Yes	Yes	Unclear	Unclear	Yes	Unclear	Yes	8
Saha; 2006	Unclear	Yes	Yes	Unclear	Yes	Yes	Yes	Yes	Yes	Unclear	Unclear	Yes	Yes	Unclear	9
Swischuk; 1986	Yes	Yes	Yes	Unclear	Yes	Yes	Yes	Yes	Yes	Unclear	Unclear	Yes	Yes	Yes	11
Toikka; 2000	Unclear	Yes	Yes	Yes	Yes	Yes	Yes	Yes	Yes	Yes	Yes	Yes	Yes	Yes	13
Tsai; 2004	Yes	Yes	Yes	Unclear	Yes	Yes	Yes	Unclear	Yes	Unclear	Unclear	Yes	Yes	Yes	10
Tzeng; 2005	Unclear	Yes	Yes	Unclear	Yes	Yes	Yes	Yes	Yes	Unclear	Unclear	Yes	Yes	Yes	10
Virkki; 2002	Unclear	Yes	Yes	Unclear	Yes	Yes	Yes	Yes	Yes	Yes	Unclear	Yes	Unclear	Yes	10
Vuori-Holopainen; 2002	Unclear	Yes	Yes	Yes	Yes	Yes	Yes	Yes	Yes	Unclear	Unclear	Yes	Yes	Yes	11

Questions: (1) Was the spectrum of patients representative of the patients who will receive the test in practice?; (2) Were selection criteria clearly described?; (3) Is the reference standard likely to correctly classify the target condition?; (4) Is the time period between reference standard and index test short enough to be reasonably sure that the target condition did not change between the two tests?; (5) Did the whole sample or a random selection of the sample, receive verification using a reference standard of diagnosis?; (6) Did patients receive the same reference standard regardless of the index test result?; (7) Was the reference standard independent of the index test (i.e. the index test did not form part of the reference standard)?; (8) Was the execution of the index test described in sufficient detail to permit replication of the test?; (9) Was the execution of the reference standard described in sufficient detail to permit its replication?; (10) 10) Were the index test results interpreted without knowledge of the results of the reference standard?; (11) Were the reference standard results interpreted without knowledge of the results of the index test?; (12) Were the same clinical data available when test results were interpreted as would be available when the test is used in practice?; (13) Were uninterruptable/intermediate test results reported?; (14) Were withdrawals from the study explained?
